# Water pollution, cholera, and the role of probiotics: a comprehensive review in relation to public health in Bangladesh

**DOI:** 10.3389/fmicb.2024.1523397

**Published:** 2025-01-14

**Authors:** Md. Rayhan Chowdhury, Ariful Islam, Valentina Yurina, Takeshi Shimosato

**Affiliations:** ^1^Graduate School of Medicine, Science and Technology, Shinshu University, Nagano, Japan; ^2^Department of Genetic Engineering and Biotechnology, University of Rajshahi, Rajshahi, Bangladesh; ^3^Department of Pharmacy, Faculty of Medicine, Universitas Brawijaya, Malang, Indonesia; ^4^Institute for Aqua Regeneration, Shinshu University, Nagano, Japan

**Keywords:** Bangladesh, water pollution, cholera, probiotics, public health

## Abstract

Cholera, a disease caused by *Vibrio cholerae*, remains a pervasive public health threat, particularly in regions with inadequate water sanitation and hygiene infrastructure, such as Bangladesh. This review explores the complex interplay between water pollution and cholera transmission in Bangladesh, highlighting how contaminated water bodies serve as reservoirs for *V. cholerae*. A key focus is the potential role of probiotics as a novel intervention approach for cholera prevention and management. Probiotics are promising as an adjunctive approach to existing therapies as they can enhance gut barrier function, induce competitive exclusion of pathogens, and modulate host immune responses. Recent probiotic advancements include engineering strains that disrupt *V. cholerae* biofilms and inhibit their virulence. Integrating probiotics with traditional cholera control measures could significantly enhance their effectiveness and provide a multifaceted approach to combating this persistent disease. This review aims to shed light on the potential of probiotics in revolutionizing cholera management and to offer insights into their application as both preventive and therapeutic tools in the fight against this enduring public health challenge.

## 1 Introduction

Water is essential for human life, and the global demand for freshwater has surged six-fold over the past century, increasing by about 1% annually since the 1980s (du Plessis, 2023). This demand strains water quality. In addition, approximately 1.6 billion people are facing economic water scarcity, and two-thirds of the world’s population are experiencing water shortages for at least 1 month annually. Industrialization, agriculture, and urbanization have polluted vital water bodies, harming health and hindering sustainable development (Ondrasek, 2013). Worldwide, it is estimated that 80% of industrial and municipal wastewater is discharged untreated into the environment, posing serious risks to both human health and ecosystems. Water-related diseases such as cholera, typhoid, polio, ascariasis, cryptosporidiosis, and various diarrheal illnesses claim the lives of approximately 3.4 million people annually in developing countries (Osiemo et al., 2019). Bangladesh is a stark example, where inadequate sanitation and insufficient wastewater treatment intensify the dual crises of water pollution and scarcity. The country, home to an intricate network of over 230 rivers, faces escalating threats from pollution driven by human activities related to water, sanitation, and hygiene. These challenges are dire, with water pollution alone contributing to 8.5% of all deaths in Bangladesh (Islam et al., 2021). Addressing these issues requires urgent, coordinated efforts to improve water management and sanitation infrastructure, not only in Bangladesh but across regions facing similar vulnerabilities. Globally, cholera remains a major health threat in over 47 countries; there are approximately 2.9 million cases of cholera and 95,000 deaths due to cholera annually (Bilal et al., 2023). In Bangladesh, cholera affects around 100,000 people and causes nearly 4,500 deaths annually. Outbreaks of the disease spike biannually, during spring and the post-monsoon season (Almagro-Moreno et al., 2015), due to seasonal factors, such as flooding, drought, and the water temperature (Ivers, 2018).

Cholera is a disease caused by the bacterium *Vibrio cholerae*. The bacterium secretes cholera toxin (CT), which causes severe watery diarrhea (Monir et al., 2022). *V. cholerae* thrives in stagnant or contaminated water, and remains highly infectious for up to 24 h after excretion. It survives the longest at water temperatures of around 30°C with 15% salinity and a pH of 8.5. The primary route of transmission is the consumption of contaminated water or food. The infectious dose of *Vibrio cholerae* ranges from 1,000 to 100 million colony-forming units, with an incubation period varying from 12 h to 5 days (Sigman and Luchette, 2012). Among the over 200 known serogroups of *V. cholerae*, the O1 and O139 serogroups have been responsible for the most recent and widespread epidemics. The O1 serogroup is further classified into the classical, El Tor, and altered El Tor biotypes (Azman et al., 2013). Cholera toxin (CT), a heat-sensitive exotoxin produced by *V. cholerae*, binds to the mucosal lining of the small intestine. This interaction disrupts cellular processes by elevating cyclic adenosine monophosphate (cAMP) levels, resulting in severe fluid and electrolyte loss (Alam et al., 2012). Additionally, *V. cholerae* employs the toxin-coregulated pilus (TcpA) as a receptor for the cholera toxin phage (CTXφ), which facilitates colonization and further exacerbates the disease (De Haan and Hirst, 2004; Boyd and Waldor, 2002). Efforts to control cholera have centered on improving water, sanitation, and hygiene (WaSH), deploying antibiotics, and expanding access to oral cholera vaccines (OCVs) (Holmgren, 2021b). However, the persistent recurrence of outbreaks in Bangladesh underscores the limitations of these measures and the urgent need for complementary strategies. Promising avenues include exploring the use of probiotics as a novel intervention to disrupt *V. cholerae* colonization and toxin production (Davies et al., 2017).

Probiotics are live microorganisms that provide health benefits when administered in adequate amounts. They have been gaining much attention for their potential to combat infectious diseases, such as cholera (Hsueh and Waters, 2019). While traditional treatments, such as antibiotics and oral rehydration therapy (ORT), remain crucial, they face limitations, including increasing antibiotic resistance (Chowdhury et al., 2022; Nalin, 2021). Thus, probiotics have been considered as a supplementary or alternative approach due to their ability to improve gut health, inhibit pathogen growth, and modulate the immune system. Strains like *Lactobacillus* and *Bifidobacterium* have shown promise in inhibiting *V. cholerae* growth and biofilm formation, which are key factors for the persistence of the pathogen (Plaza-Diaz et al., 2019). These probiotics produce acidic byproducts that lower the pH level, creating a hostile environment for *V. cholerae* (Alamdary and Bakhshi, 2020). They also boost host immune responses by enhancing immunoglobulin A (IgA) production, which strengthens the mucosal barrier against CT (Gou et al., 2022). Moreover, probiotics, such as *Lactobacillus rhamnosus* GG, have been shown to reduce the levels of proinflammatory cytokines, such as interleukin-8 (IL-8), helping to maintain intestinal integrity and prevent severe inflammation during *V. cholerae* infection (Nandakumar et al., 2009). Engineered probiotics, such as *Lactococcus lactis*, have been designed to detect and disrupt *V. cholerae* quorum sensing, and thereby reduce its virulence (Mao et al., 2018). In addition, recombinant *Escherichia coli* strains expressing GM1 ganglioside mimics have been shown to neutralize CT (Yu et al., 2016). Additionally, probiotics can enhance the effectiveness of OCVs by promoting a favorable gut environment (Amdekar et al., 2010). Commensal bacteria like *Ruminococcus obeum* can interfere with *V. cholerae* quorum sensing and further shows the potential of using probiotics to modulate the gut microbiome in favor of the host and against the pathogen (Jimenez and Sperandio, 2019). These multi-faceted benefits—from antimicrobial action to immune support—highlight the potential of probiotics in cholera management, particularly in regions where conventional methods are limited. This review examines the water pollution situation in Bangladesh, its link to cholera outbreaks, and the role of probiotics in mitigating the impact of the disease.

## 2 Water pollution in Bangladesh

**FIGURE 1:**
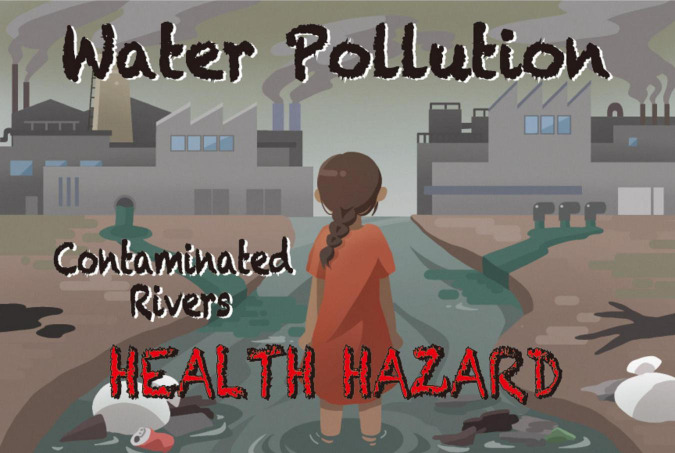
Pathways of water pollution leading to health impacts in Bangladesh. The pollutants enter water bodies, leading to contaminated rivers, human exposure, and adverse health effects.

Bangladesh is a nation with a vast river system and a dense population, and water contamination is a major environmental and public health issue in the country (Figure 1). The country’s water resources, including over 230 rivers, are under significant threat due to both natural and human activities. Severe contamination is observed in both surface and groundwater sources, posing serious risks to public health, the environment, and the economy (Uddin and Jeong, 2021). There are significant regional variations in water quality in Bangladesh. In this section, data on the water pollution in key regions of Bangladesh are summarized with a focus on the dissolved oxygen (DO), biochemical oxygen demand (BOD), chemical oxygen demand (COD), and heavy metal levels.

Rivers near Dhaka city, including the Buriganga, Turag, and Shitalakshya rivers, are severely polluted due to industrial activities and urban runoff. The Buriganga river has critically low DO levels (0.9–2.8 mg/L) and high COD levels (140–800 mg/L) due to untreated effluents (Sarkar et al., 2019). Similarly, the Turag river has near-zero DO levels, COD levels of 102–475 mg/L, and high BOD levels (Ahmed et al., 2016). The Shitalakshya river shows near-zero DO levels, and elevated BOD and COD levels, especially during the dry season, due to industrial effluents (Mourshed et al., 2017). In northern Bangladesh, rivers, such as the Teesta, Korotoa, and Atrai rivers, are impacted by agricultural runoff and urban wastes. The Teesta river has high levels of heavy metals, such as cadmium, iron, and manganese, exceeding the standards of the World Health Organization and the Food and Agriculture Organization of the United Nations (Chettri and Joshi, 2022). The Korotoa river has high concentrations of chromium and cadmium, and DO levels below 4 mg/L (Hassan et al., 2024). The Atrai river has moderate-to-high pollution levels due to seasonal agricultural and urban waste discharge (Anik et al., 2022). Rivers in the southern coastal regions, including the Karnaphuli and Meghna rivers, are polluted from industrial discharges and agricultural runoff (Gani et al., 2023). The Karnaphuli river is polluted from shipbreaking yards and industries, and has high levels of heavy metals, such as lead, cadmium, and mercury (Chowdhury et al., 2024). The Meghna river is contaminated by upstream pollution sources; it has COD levels of 20.84–114.6 mg/L, and high levels of heavy metals, such as iron and chromium, that exceed the safe limits (Flura et al., 2016). The Ganges and Padma rivers in western Bangladesh are important as sources of water for drinking and for agriculture, but they are becoming increasingly polluted. The Ganges river has moderate DO levels and elevated BOD levels as well as high concentrations of arsenic and lead (Mukherjee et al., 1993). The Padma river shows fluctuating water quality, with occasionally high coliform levels and suboptimal DO, BOD, and COD levels that reflect agricultural runoff and industrial discharges (Flura et al., 2016). The groundwater in rural Bangladesh is heavily contaminated with arsenic; the concentration often exceeds 10 μg/L, and reaches up to 500 μg/L in some areas. This contamination affects millions of people who rely on tube wells for drinking water, and leads to widespread health issues, such as arsenicosis and cancer (Chakraborti et al., 2010). Overview of key water quality parameters in the table below showed the actual water quality in Bangladesh (Table 1).

**TABLE 1:** Overview of key water quality parameters for major rivers in Bangladesh.

River	pH	EC (μ S/cm)	DO (mg/L)	BOD (mg/L)	COD (mg/L)	Coliform (CFU/ml)	TDS (mg/L)	Cl (mg/L)	NO3- (mg/L)	NO2- (mg/L)	PO43- (mg/L)	Research Year	References
Brahmaputra	7.75	351.12	4.47	1.02	–	–	178.54	–	–	–	–	2013–2014	Uddin and Jeong, 2021
	7.12–8.9	76–190	4.3–8.3	–	–	–	34.5–80	14.2–85.2	1.13–1.30	0.01–0.03	–	2014–2015	
	7.45–7.65	348–355	3.20–3.70	3.6–4.2	–	–	205–220	–	–	–	–	2016	
Turag	7.4–7.5	566–593	–	–	25–45	220–340	402–419	–	1–2	0.1	1.5–2	2010	
	6.6–7.98	160–1,107	0.11–6.8	10–180	21–220	–	100–580	–	–	–	–	2010	
	7.73	0.37	296.91	251.83	–	–	82.83	56.38	190.33	80.06	–	2011	
	6.9–9.1	790–2,850	0.45–3.20	56–179	5–177	–	650–1,510	–	–	–	–	2011–2012	
	6.18–7.46	35–150	0.6–3.9	0.4–1.9	–	–	203–902	–	–	–	–	2013	
	7.40–7.79	276–303	–	–	–	–	181.7–194.5	–	–	–	–	2014	
	5.86–7.28	354.5–488.75	3.49–5.2	42.34–55.92	102.6–181.7	–	109.61–196.7	–	–	–	–	2014–2015	
	7.5–8.2	0.0325–0.0535	–	–	–	–	225.4–356.2	–	–	–	–	2015	
	–	–	–	–	–	–	–	1–4.87	0.218–1.25	9.9–26.10	–	2015	
	6.35–6.75	1,850–2,120	3.75–4.10	2.90–3.30	–	–	3,460–4,145	–	–	–	–	2015	
	7.24–7.61	425–2,277	1.22–3.66	−2.44 to 0.86	–	–	239–1,349	–	–	–	–	2016	
	–	340–610	2.32–6.28	13–73	–	–	582–655	–	–	–	–	2016	
Shitalakshya	–	–	0.07–7.52	0.4–28.8	1–61	–	–	–	0.2–1.8	–	0.08–2.8	2008	
	7.2–9	503–1,672	120	180	357–1,118	–	1	0.02–1	0.15–2	–	–	2010	
	7.54	0.46	488.58	519.32	–	–	56.42	55.33	171.67	33.29	–	2011	
	7.22–7.32	–	2.32–3.08	30–140	130–280	–	743–858	–	–	–	–	2011–2012	
	6.5–8.3	720–1,920	0.6–3.8	44–146	14–172	–	475–1,180	–	–	–	–	2011–2012	
	6.9–8	121–1,167	0.5–3.5	–	80–480	–	80–754	–	–	–	–	2012–2013	
	6.7–7.25	986–2,321	1.2–3.12	25.12–35.12	89.72–118.1	–	639.1–1,171	–	–	–	–	2014–2015	
	7.4–7.7	443–1,175	1.3–2.63	0.55–1.3	–	–	269–573	–	–	–	–	2015	
	6.5–7.6	108–478	6–12	–	–	54–245	3.54–9.91	–	–	–	–	2017–2018	
Meghna	6.07–8.01	61.30–182.20	4.66–8.35	1.20–10.10	20.84–114.62	–	–	–	–	–	–	2014	
Surma	5.86–6.86	759–850	3.5–7.2	0.6–1.8	1–2.6	11–182.5	38.46–1,478.9	–	–	–	–	2001–2003	
Karnaphuli	6.36–9.86	90–45,600	0–7.91	0.21–9.17	11.39–179.87	–	45–20,000	2.09–13,147.70	0–1.63	0–5.18	–	2008–2009	
	6.2–7	552–31,340	0.10–3	160–370	350–755	–	292–18,530	–	–	–	–	2003; 2008	
	–	–	–	15.50–630	20.80–832	–	–	–	0.20–1.50	0.10–0.80	0.50–45	2013	
	6.8–7.9	3,020–20,500	1.50–5.20	168–380	322–765	–	1,963–15,262	13.88–41.35	–	–	–	2016	
Halda	7.03–8.60	72–414	3.02–9.90	0.70–5.08	14.78–49.28	–	30–200	2.41–73.50	0.00	0–0.87	–	2008–2009	
	6.3–7.3	110–524	0.93–5.15	30–545	43–983	–	–	12–56	–	–	–	2015–2016	
	7.10–8.80	–	3.35–4.70	0.055–5	–	–	–	8.4–69.30	0.12–3.10	–	0.06–0.16	2016	
	7.08–7.65	96.1–218	5.90–8.40	0.30–2.80	24–96	–	45.5–104.1	25–54.5	–	–	0.13–0.38	2018	
Sangu	7.66	270.90	5.83	2.24	–	–	135.32	14.35	0.25	0.01	–	2008–2009	
Kaptai lake	7.90	85.87	6.85	1.27	–	–	41.33	3.96	0.44	0.01	–	2008–2009	
Matamuhuri	7.71	237.50	5.64	3.60	–	–	118.55	13.88	0.00	0.00	–	2008–2009	
Naf	7.73	49,300.00	7.56	6.92	–	–	24,700.00	21,720.92	0.00	0.00	–	2008–2009	
Bhairab	6.56–8.07	173–1,329	3.5–6.5	–	–	–	–	15.37–460.85	0.66–14.47	–	–	2015–2016	
Rupsa	7.0–8.18	193–4,120	4.0–5.6	–	–	–	–	22.25–131,1.65	1.52–30.72	–	–	2015–2016	
	8.1–9.0	13,730–20,470	–	–	–	–	6,900–11,000	444–724	–	–	–	2016–2017	
Mayur	6.88–7.34	770–1,670	–	–	–	–	600–1,100	0.018–0.069	0.979–23.887	–	–	2012	

### 2.1 Types of water pollution in Bangladesh

Water pollution is the contamination of water bodies—rivers, lakes, seas, and groundwater—primarily caused by human activities. It occurs when harmful substances, such as chemicals, wastes, and pathogens, are introduced into the water, rendering it unsafe for consumption, disrupting ecosystems, and degrading the environment (Chakraborti et al., 2010). In Bangladesh, the water pollution can be categorized into different types, organic, inorganic, chemical, and pathogenic.

Organic pollution is mainly caused by untreated domestic sewage and industrial effluents that are high in biodegradable matter. As the biodegradable matter decomposes, the DO level decreases, which is harmful to aquatic life. Indicators like the COD and BOD are used to measure this type of pollution. Rivers like the Buriganga, Turag, and Shitalakshya rivers have critically low DO levels, indicating severe organic contamination (Islam and Azam, 2015). Inorganic pollutants, such as salts and metals, persist in the environment and cannot be biodegraded. In Bangladesh, industrial discharges, agricultural runoff, and natural leaching are the primary sources of inorganic pollutants. Key contaminants, such as arsenic and iron, are found in groundwater, and arsenic poses a significant public health risk (Rahman et al., 2021). Chemical pollution primarily arises from industrial processes, particularly in the textile, dyeing, and leather industries. Untreated chemicals, including dyes, acids, and heavy metals, are released into rivers, causing severe water degradation. The Karnaphuli river near Chittagong is notably affected by these pollutants (Islam et al., 2017). Pathogenic pollution occurs when harmful microorganisms, such as bacteria, viruses, and protozoa, contaminate water. Improper sewage disposal, human wastes, and inadequate sanitation contribute to this problem. Waterborne diseases, such as cholera, diarrhea, and dysentery, are prevalent in Bangladesh, particularly in rural areas with limited access to clean water (Table 2; Knappett et al., 2011). Agriculture, while vital to Bangladesh, contributes significantly to water pollution. Fertilizers and pesticides, such as carbamates and organophosphates, wash into water bodies, contaminating surface water and groundwater. This runoff bioaccumulates in the food chain, posing risks to ecosystems and human health (Chopra et al., 2011). Thermal pollution occurs when industries discharge heated water into natural water bodies, raising the water temperature and disrupting ecosystems; this is particularly harmful to temperature-sensitive species. In Bangladesh, this issue is seen in regions where industrial plants discharge heated cooling water (Kibria and Yousuf Haroon, 2017). Improper disposal of plastic and solid wastes also contributes heavily to water pollution. Rivers and canals in urban areas like Dhaka are clogged with non-biodegradable wastes, worsening floods during monsoons and harming aquatic life. Plastic accumulation also creates breeding grounds for disease vectors (Mourshed et al., 2017).

**TABLE 2:** Bacteria in Bangladesh water and their health hazards.

Bacteria	Possible health effects	References
*Aeromonas hydrophila*	Septicemia, gastroenteritis	Hasan et al., 2019
*Enterobacter aerogenes*	Urinary and respiratory tracts infection	
*Enterococcus species*	Cause infection in urinary tract	
*Escherichia coli*	Cause food poisoning, gastroenteritis, urinary tract infections	
*Klebsiella species*	Urinary tract, respiratory tract, lung, wound infections	
*Listeria species*	Meningitis, endocarditis	
*Salmonella species*	Typhoid	
*Shigella species*	Abdominal pain, tenesmus, watery diarrhea	
*Staphylococcus species*	Superficial skin lesions, food poisoning	
*Vibrio* spp.	Cholera	
*Bacillus* sp.	Food Poisoning	Mina et al., 2018
*Cardiobacterium* sp.	Endocarditis, bacteremia	
*Corynebacterium* sp.	Diphtheria, skin infections, bacteremia, urinary tract infections, respiratory infections, wound infections	
*Clostridium* sp.	Botulism, tetanus, gas gangrene, food poisoning, Pseudomembranous colitis, necrotizing enterocolitis	
*Lactobacillus* sp.	Bacteremia, endocarditis, urinary tract infections, liver abscess, dental caries	
*Micrococcus* sp.	Bacteremia, endocarditis, pneumonia, skin infections, meningitis	
*Campylobacter* spp.	Campylobacteriosis, gastroenteritis, Guillain-Barré syndrome, bacteremia, reactive arthritis, meningitis, hepatitis	Saima et al., 2023
*Campylobacter coli*	Campylobacteriosis, gastroenteritis, bacteremia, reactive arthritis, Guillain-Barré syndrome	
*Campylobacter jejuni*	Campylobacteriosis, gastroenteritis, irritable bowel syndrome	

### 2.2 Major sources of water pollution in Bangladesh

Water pollution is a major environmental challenge in Bangladesh that threatens public health, aquatic ecosystems, and environmental sustainability. The primary sources of the pollution are diverse, and are driven by rapid industrialization, urbanization, and agricultural practices (Singh et al., 2004). The key sources of water pollution in Bangladesh are discussed below with examples from various studies and reports.

Rapid industrialization, especially in textiles, dyeing, leather, and pharmaceuticals, has led to large volumes of untreated wastewater being discharged into water bodies. The textile sector alone produced around 217 million cubic meters of wastewater in 2016, and the figure was projected to rise to around 349 million cubic meters by 2021 (Hossain et al., 2018). Such wastewater containing dyes, chemicals, and/or heavy metals, such as chromium, cadmium, and lead, severely contaminates water sources around Dhaka and Chittagong (Mina et al., 2018). The tannery sector in Hazaribagh also contributes to water pollution by releasing hazardous chemicals, such as sulfides and acids (Anawar et al., 2000). Pharmaceutical and chemical industries further pollute aquatic ecosystems, as seen in the heavily impacted Karnaphuli river (Chowdhury et al., 2024).

Urbanization and population growth have led to increases in household and municipal wastes, much of which ends up in water bodies due to poor waste management. Inadequate sewage treatment, such as at the Pagla Sewage Treatment Plant, results in raw sewage being discharged into rivers like the Buriganga and Shitalakshya rivers. Raw sewage contains organic matter and pathogens that lead to the spread of waterborne diseases, such as cholera (Majed and Islam, 2022). Urban solid wastes, including plastics, are frequently dumped into water bodies, causing blockages and pollution. The Buriganga river is biologically inactive in some sections due to severe solid waste pollution (Reza and Yousuf, 2016; Karn and Harada, 2001).

Agricultural runoff from excessive fertilizer and pesticide use is a major source of non-point source water pollution. Fertilizers, pesticides like DDT and carbofuran, and contaminants in irrigation water lead to bioaccumulation in water bodies, which poses risks to aquatic life and human health (Chhabra, 2021). The healthcare sector also contributes to water pollution by disposing of pharmaceuticals, disinfectants, and biological wastes. Many of Bangladesh’s 600 hospitals lack proper waste disposal systems, and as a result, toxic substances enter water bodies and pose health risks (Hassan et al., 2008).

The shipbreaking industry in coastal Chittagong is another significant source of pollution; it releases heavy metals, asbestos, and oil residues into the sea, contaminating the Karnaphuli river and nearby waters. Ships also discharge bilge water containing hazardous substances, which further degrade the water quality and marine ecosystems (Islam et al., 2013). Natural sources, such as arsenic and iron, also contribute to water pollution. The groundwater in Bangladesh contains high levels of arsenic, affecting millions of people who rely on tube wells for drinking water. Long-term exposure to arsenic can lead to severe health issues, including skin lesions and cancers (Huq et al., 2020).

## 3 Linking water pollution to cholera outbreaks

Water pollution and cholera are closely linked, particularly in developing countries like Bangladesh, where access to clean water is limited. Frequent flooding and widespread contamination of water bodies by *V. cholerae*, the bacterium responsible for cholera, contribute significantly to the transmission of the disease (Pandey et al., 2014). This section explores how water pollution contributes to cholera outbreaks, with examples from recent studies.

Cholera is primarily transmitted through the ingestion of water contaminated with *V. cholerae*. The bacteria is naturally present in freshwater and brackish water, and it often attaches to plankton, particularly zooplankton like copepods, which act as reservoirs (Lutz et al., 2013). The zooplankton play a crucial role in facilitating the survival of the bacteria between epidemics, especially during periods of high plankton density following phytoplankton blooms (de Magny et al., 2011). Cholera spread is a complex interplay of human activity, travel, and hydroclimatic processes, affecting the distribution, growth, and incidence of *V. cholerae* in aquatic ecosystems, and can be improved through epidemiological research and environmental predictive modeling (Usmani et al., 2021). Studies have also shown that the survival of *V. cholerae* in environmental reservoirs is influenced by the water temperature and salinity, and the presence of organic wastes (Huq et al., 1984; McCarthy, 1996). In Bangladesh, a strong correlation between environmental conditions and cholera outbreaks has been documented, especially during the monsoon season when increased rainfall leads to sewage mixing with drinking water, which increases the risk of cholera (Akanda et al., 2013). Conversely, during the dry season, increased salinity also creates favorable conditions for *V. cholerae* proliferation (Jutla et al., 2013). Studies have further demonstrated a link between water pollution and the incidence of cholera, and both natural and human-induced changes in water quality in Bangladesh have been linked to the persistence of *V. cholerae* in polluted and stagnant waters. Water of poor quality, characterized by high pollution levels, low oxygen levels, and pH imbalances, provides ideal conditions for *V. cholerae* survival (Nguyen et al., 2023). For instance, in Bakerganj, Bangladesh, a study found a strong association between water pollution parameters (such as water temperature, salinity, and conductivity) and the incidence of cholera. A six-week lag was observed between peaks in water temperature and cholera cases, highlighting the role of environmental factors in cholera transmission (Huq et al., 2005). In addition, moderate salinity was found to support bacterial growth, especially in coastal areas during the dry season (Grant et al., 2015). Data from the Matlab Demographic Surveillance Site have also highlighted the role of environmental factors, such as elevated water temperatures and salinity, in promoting *V. cholerae* growth and cholera outbreaks.

## 4 Current management strategies for cholera in Bangladesh

In Bangladesh, cholera remains a significant public health issue that is driven by environmental factors, the population density, and the limited access to clean water and sanitation. Effective management strategies are crucial for controlling outbreaks. Bangladesh has developed a robust surveillance system, including hospital and community monitoring, particularly during the peak seasons (Hegde et al., 2021). Environmental testing helps predict outbreaks, and both passive and active surveillance enable early interventions (Azman et al., 2015). Once an outbreak is detected, rapid response teams provide medical care, distribute oral rehydration solutions (ORS), and educate communities on prevention measures (Zohura et al., 2022). ORS are highly effective for mild dehydration, while intravenous fluids, such as Dhaka solution, are used for severe cases, reducing the fatality rates (Davies et al., 2017). Antibiotics are prescribed for severe cases, but due to concerns of antimicrobial resistance, they need to be used prudently and require careful monitoring (Leibovici-Weissman et al., 2014). In addition, community engagement is important and can be promoted through public health campaigns focusing on hygiene practices, safe water use, and ORS preparation, particularly in high-risk areas (Khan et al., 2019). Bangladesh’s vaccination strategy combines reactive and preventive approaches to control cholera. Reactive vaccinations contain disease outbreaks, while preventive vaccinations target high-risk populations before outbreaks. This dual approach ensures effective use of vaccines mediated successfully by WaSH (Ronsmans et al., 1988; Chowdhury et al., 2023). Bangladesh employs both reactive and preventive vaccination strategies; however, logistical challenges, such as maintaining the cold chain for vaccine storage, remain (Dimitrov et al., 2014; Uddin et al., 2014). WaSH interventions, including clean water provision, latrine construction, and hygiene education, are essential for cholera prevention, and they have been supported by local governments and non-governmental organizations (Lantagne and Yates, 2018). Bangladesh is also a hub for cholera research, with the icddr,b, an international health research organization located in Dhaka, providing valuable insights into vaccine efficacy, rehydration solutions, and the role of micronutrients, such as zinc (Holmgren, 2021a). Technological innovations, such as the mHealth Diarrhea Management (mHDM) platform, help guide healthcare providers with evidence-based treatments, enabling better antimicrobial stewardship (Chowdhury et al., 2022). Prophylactic measures, including OCVs and selective chemoprophylaxis, are used to prevent the spread of cholera, especially among the close contacts of confirmed cases (Weil et al., 2014). Zinc and vitamin A supplementation are recommended for children under five years old to manage cholera, reduce diarrhea severity, enhance immune response, and promote intestinal lining recovery, as part of standard pediatric cholera management in Bangladesh (Albert et al., 2003; Liberato et al., 2015). New therapies, such as probiotics and phage therapy, are being explored as adjunct treatments, and they appear promising as solutions for dealing with antibiotic-resistant cholera strains and reducing the bacterial load in patients (Hsueh and Waters, 2019; Bhandare et al., 2019).

The evaluation of these strategies underscores a prioritized approach to cholera management, tailored to the severity of cases and the specific implementation context. Oral rehydration salts (ORS) and intravenous fluids remain the cornerstone of cholera treatment, particularly in acute cases, due to their immediate life-saving potential. In parallel, vaccination campaigns and WaSH interventions are indispensable for long-term prevention. However, these measures face persistent logistical challenges, including maintaining vaccine cold chains and ensuring the development of adequate sanitation infrastructure. Probiotics are emerging as a promising adjunctive therapy, offering significant potential in addressing antibiotic-resistant *Vibrio cholerae* strains. Incorporating probiotics into existing management frameworks could fill critical gaps in treating resistant cases, thereby bolstering Bangladesh’s public health resilience. To fully harness this potential, further research is needed to evaluate the integration of probiotics with established therapies. Such efforts could pave the way for sustainable and effective alternatives to conventional antibiotics, strengthening both immediate treatment outcomes and long-term disease control.

## 5 Probiotics: an overview

Probiotics, derived from the Greek words “pro” (for) and “bios” (life), refer to live microorganisms that provide health benefits when consumed in sufficient amounts. Unlike antibiotics, which aim to kill harmful bacteria, probiotics work to enhance or restore the microbial balance in the host (Gou et al., 2022). The most widely accepted definition of probiotics comes from the Food and Agriculture Organization and the World Health Organization of the United Nations, who described them as “live microorganisms which, when administered in adequate amounts, confer a health benefit on the host” (Reid et al., 2005). According to this definition, probiotics need to be alive, administered in proper doses, and scientifically proven to offer health advantages. Similarly, the following criteria have been proposed for microorganisms to be classified as probiotics: they must be alive when administered, present in sufficient quantities, have demonstrated health benefits, and be safe for consumption (Kaur et al., 2021).

The concept of probiotics dates back to the early 20th century, when Nobel laureate Élie Metchnikoff suggested that the consumption of fermented milk could promote longevity by improving gut health due to the presence of beneficial bacteria in the milk. His work laid the foundation for modern probiotic research. Probiotics have since been shown to positively affect digestive health, boost immune function, and influence mental health via the gut-brain axis (Tong et al., 2020).

Probiotics predominantly consist of bacteria that are commonly of the genera *Lactobacillus*, *Bifidobacterium*, *Streptococcus*, and *Bacillus*, although some yeast strains, such as *Saccharomyces boulardii*, also exhibit probiotic properties (Gupta and Garg, 2009). *Lactobacillus* species are known to improve gut health, enhance lactose digestion, prevent diarrhea, and support respiratory health (Reid, 1999). *Bifidobacterium* species are known to play a crucial role in maintaining a healthy gut environment, preventing infections, and supporting immune functions (Turroni et al., 2022). These bacteria promote gut health by producing short-chain fatty acids that help maintain the integrity of the intestinal lining. Yeast probiotics, particularly *S. boulardii*, have gained attention for their effectiveness in treating gastrointestinal disorders, such as antibiotic-associated diarrhea and infections caused by *Clostridium difficile*. *S. boulardii* can withstand the acidic conditions of the stomach, temporarily colonize the gut, and enhance immunity and the gut barrier (Czerucka et al., 2007).

Beyond gut health, probiotics have been linked to mental health benefits through the gut-brain axis (Lenoir-Wijnkoop et al., 2007). Strains of *Lactobacillus* and *Bifidobacterium* have been shown to produce neurotransmitters, such as gamma-aminobutyric acid and serotonin, which regulate mood. The results of clinical studies suggest that probiotics can help alleviate symptoms of anxiety, depression, and stress, and may therefore provide a potential therapeutic approach for mental health (Bermúdez-Humarán et al., 2019).

Probiotics also contribute to metabolic health by addressing imbalances in the gut microbiota that are linked to obesity and type 2 diabetes. They help improve insulin sensitivity, reduce inflammation, and promote weight management. For instance, *Bifidobacterium lactis* has been shown to reduce body fat and improve glucose tolerance in animal models, indicating its potential for managing metabolic disorders (Nagpal et al., 2016).

Moreover, probiotics play an essential role in immune health. They enhance immune responses by increasing the IgA levels, boosting natural killer cell activity, and improving macrophage functions, which can help protect against infections, and possibly even against cancer. Certain strains, such as *Lactobacillus casei*, have demonstrated the ability to enhance immune responses to respiratory infections (Śliżewska et al., 2020). These findings highlight the broad potential applications of probiotics for promoting health.

### 5.1 Mechanism of action of probiotics

Probiotics exert beneficial effects through several mechanisms, including the modulation of immune responses, enhancement of gut barrier function, competitive exclusion of pathogens, production of antimicrobials, modulation of the gut microbiota composition, interaction with the gut-brain axis, and effects on metabolic health. They modulate immune responses by balancing pro- and anti-inflammatory cytokines, enhancing IL-10 production, and reducing the levels of tumor necrosis factor-α and IL-6, which drive conditions like inflammatory bowel disease (Cristofori et al., 2021). Probiotics also strengthen the intestinal barrier by promoting the production of proteins like occludin and claudin, which prevent pathogen translocation (Castro-Herrera et al., 2020). Through competitive exclusion, probiotics compete with pathogens for adhesion sites, and they also produce antimicrobial substances, including bacteriocins and organic acids, which inhibit pathogen growth (Tejero-Sariñena et al., 2012; Chen et al., 2018). Furthermore, probiotics influence the composition of the gut microbiota and support gut health by promoting the growth of beneficial bacteria and suppressing the growth of harmful species, such as *Clostridium* species (Wieërs et al., 2019). Additionally, they interact with the gut-brain axis to induce the production of neurotransmitters like gamma-aminobutyric acid, which can modulate brain function, offering potential therapeutic benefits for mental health conditions (Tette et al., 2022). In terms of metabolic health, probiotics help regulate energy metabolism, improve glucose tolerance, and enhance insulin sensitivity, primarily through the production of short-chain fatty acids (Everard and Cani, 2014). Moreover, probiotics interact with the enteric nervous system to influence gut motility and secretion, which is beneficial for conditions like irritable bowel syndrome (Kunze et al., 2009). They also enhance mucosal immunity by increasing the production of secretory IgA, which strengthens the host defense against pathogens (Azad et al., 2018). Probiotics can regulate inflammatory pathways, including the nuclear factor kappa B signaling pathway, and thereby reduce inflammation in conditions like inflammatory bowel disease (Versalovic et al., 2008). Probiotics can also protect against enteric infections by preventing pathogen adhesion and enhancing immune responses (Resta-Lenert and Barrett, 2003), which further highlights the therapeutic potential of probiotics.

### 5.2 Current research on probiotics in cholera control

In addition to the traditional cholera control measures, probiotics have emerged as a promising adjunctive treatment. Probiotics can inhibit *V. cholerae* colonization and neutralize toxins through competitive exclusion, the production of antimicrobial compounds, enhancement of intestinal barrier function, and modulation of the host immune response, and thereby prevent the spread of the disease (Hsueh and Waters, 2019; Chowdhury et al., 2022). This section reviews evidence from various studies that highlight the efficacy of probiotics for preventing and managing cholera infections.

Maintaining a balanced gut microbiota is crucial for limiting the colonization and virulence of *V. cholerae*. Research has demonstrated that the gut microbiota can affect *V. cholerae* by modulating chemical signaling pathways, such as those involved in quorum sensing and bile acid metabolism. By influencing these pathways, the commensal bacteria can alter the ability of *V. cholerae* to express virulence factors and establish an infection. The gut microbiota also competes with *V. cholerae* for nutrients, which can limit the pathogen’s growth. The interactions between *V. cholerae* and the gut microbiota are complex and involve various microbial and host factors. Probiotics can modulate the gut microbiota to enhance host resistance against *V. cholerae* colonization. For example, *R. obeum* has been identified as a key bacterium that increases in abundance during recovery from cholera; it was found to restrict *V. cholerae* colonization by producing autoinducer-2, which interferes with the pathogen’s quorum-sensing pathways and downregulates the expression of virulence genes. This natural antimicrobial effect suggests that promoting the growth of such beneficial bacteria through probiotic supplementation could serve as a strategy for preventing and/or mitigating *V. cholerae* infections (Hsiao et al., 2014).

The ability of *V. cholerae* to form biofilms is a critical factor for its survival and persistence, both in aquatic environments and the human gut. Probiotics have been shown to have significant potential in disrupting *V. cholerae* biofilms. For instance, lactobacilli isolated from fecal samples have been shown to inhibit biofilm formation and to disperse formed biofilms by more than 90%. This effect is partly due to the production of acidic byproducts by the lactobacilli; the acidic byproducts alter the pH and disrupt biofilm matrices. Mao et al. (2018) demonstrated that the oral administration of *Lactococcus lactis*, a widely used fermentative bacterium, significantly reduced the intestinal burden of *Vibrio cholerae* in infected newborn mice. By producing lactic acid, *L. lactis* enhanced colonization resistance and improved survival rates. Furthermore, an engineered strain of *L. lactis* was developed to detect quorum-sensing signals from *V. cholerae* and produce an enzyme reporter for stool-based diagnostics, opening new avenues for innovative surveillance and intervention strategies (Mao et al., 2018). In addition to *L. lactis*, *Saccharomyces boulardii*, a probiotic yeast, has shown potential in binding and neutralizing cholera toxin *in vitro*. Similarly, *Lactobacillus rhamnosus GG* and *Bifidobacterium longum* demonstrated significant toxin removal capabilities, neutralizing 68 and 59% of cholera toxin, respectively, under laboratory conditions. These findings highlight the importance of live probiotic cultures in cholera management, as these effects were both concentration-dependent and absent in non-viable cells or their supernatants. Probiotic activity extends beyond toxin neutralization. Certain *Lactobacillus* strains have shown remarkable ability to inhibit biofilm formation by *V. cholerae* and disperse preformed biofilms, achieving inhibition rates exceeding 90% in some cases. These effects were strain-specific and pH-dependent, with biofilm dispersal activity diminishing under neutralized pH conditions. Given that the physiological pH of intestinal biofilms is slightly acidic, strains exhibiting dispersive activity under such conditions are particularly promising for therapeutic applications (Kaur et al., 2018). These findings underscore the multifaceted potential of probiotics in cholera management, ranging from toxin neutralization and biofilm disruption to diagnostic innovation. Future research should prioritize the identification of robust, pH-tolerant strains with clinically significant activity, paving the way for integrative approaches in combating this persistent global health challenge.

*L. lactis* expressing *V. cholerae* antigens can stimulate both systemic and mucosal immune responses, providing enhanced protection against cholera (Lei et al., 2011). This dual role of probiotics in competing directly with pathogens and in priming the immune system makes them valuable tools for both prevention and treatment. Engineered probiotics also hold promise as diagnostic tools. For example, *L. lactis* was engineered to detect quorum-sensing signals from *V. cholerae* and to express a reporter enzyme that can be detected in fecal samples (Amrofell et al., 2020). This ability to detect and respond to specific pathogenic signals may enable a targeted approach for controlling *V. cholerae* infections in real time. Another promising approach involves engineering probiotics to neutralize CT directly. In one study, recombinant *E. coli* strains that express GM1 ganglioside mimics on their surfaces were developed; these mimics could bind to CT and neutralize its harmful effects (Yu et al., 2016). The GM1-expressing *E. coli* conferred significant protection against fatal *V. cholerae* infections in infant mice, even when they were administered post-infection. The high affinity of these recombinant probiotics for CT highlights their potential usefulness as therapeutic agents in both the prevention and treatment of cholera, offering an alternative to traditional antibiotic therapies (Focareta et al., 2006). In another similar study, probiotics engineered to detect and respond to *V. cholerae* as an innovative therapeutic strategy were shown to produce antimicrobial compounds that inhibit *V. cholerae* and disrupt its quorum-sensing mechanisms, which are essential for its virulence and for biofilm formation (Cruz et al., 2022).

Probiotics can influence *V. cholerae* through metabolic interactions within the gut environment. Studies using zebrafish models have shown that *E. coli* strains capable of metabolizing glucose into acidic byproducts significantly reduce *V. cholerae* colonization. The resulting acidic environment inhibits *V. cholerae* growth, and is a mechanism by which the metabolic byproducts of commensal or probiotic bacteria can create unfavorable conditions for the pathogen (Nag et al., 2018). The use of multiple probiotic strains together can result in enhanced antimicrobial effects against *V. cholerae*. For example, a combination of *Leuconostoc mesenteroides* and *Bacillus subtilis* in a malted ragi food product was found to significantly inhibit *V. cholerae* growth, biofilm formation, and adhesion to extracellular matrices. The synergistic action of these probiotics not only enhanced their antimicrobial activity, but also increased the nutritional value of the food, suggesting that functional foods incorporating probiotics could serve dual roles in nutrition and disease prevention (VidyaLaxme et al., 2014). Combining probiotics with conventional cholera treatments, such as ORT and vaccines, could also enhance the overall treatment efficacy. For instance, studies have shown that probiotics capable of acidifying the gut environment can work synergistically with glucose-based ORT to inhibit *V. cholerae* growth (Basumatary et al., 2021).

Additionally, probiotics have been observed to modulate gut microbial communities in ways that may support the efficacy of OCVs. By promoting a healthier gut microbiota, probiotics can enhance the host immune response to vaccination, and also potentially enhance host defenses against cholera infection (Sit et al., 2019). For example, *Bifidobacterium breve* BBG-01 was studied for its potential to boost the immunogenicity of an inactivated cholera vaccine. Although the enhancement of vaccine-induced immune responses was not significant, the probiotic did modify the gut microbiota by increasing the *Bifidobacterium* and reducing the *Enterobacteriaceae* counts. This shift toward a healthier gut microbiota may promote the overall effectiveness of vaccination by creating a less favorable environment for *V. cholerae* colonization and enhancing the baseline immunity of the host (Matsuda et al., 2011). Some other studies have explored using probiotics as a vaccine delivery system. For example, *L. lactis* expressing *V. cholerae* antigens was found to induce strong mucosal and systemic immunity, and may represent a novel approach to vaccination. The dual role of probiotics as both a vaccine adjuvant and a protective agent against cholera highlights their usefulness and versatility in cholera control strategies (Zamri et al., 2012).

Probiotics can also modulate the host inflammatory response to *V. cholerae* infection to provide a protective effect. For instance, *L. rhamnosus* GG has been shown to reduce the expression of IL-8 and other chemokines in human intestinal epithelial cells exposed to *V. cholerae*. By attenuating the inflammatory response, such probiotics can help maintain the gut integrity and reduce the damage caused by excessive inflammation, which is often a hallmark of severe cholera. This anti-inflammatory effect is yet another layer of protection conferred by probiotics during cholera infection (Nandakumar et al., 2009). Additionally, studies have shown that the ingestion of yogurt containing *Lactobacillus acidophilus* and *Bifidobacterium* enhances IgA responses to CT in mice. IgA plays a key role in neutralizing toxins and preventing them from adhering to and penetrating the intestinal epithelium, thereby enhancing mucosal immunity. The use of dietary probiotics to enhance mucosal immunity could therefore be an effective strategy for bolstering the body’s natural defenses against cholera (Tejada-Simon et al., 1999).

**FIGURE 2:**
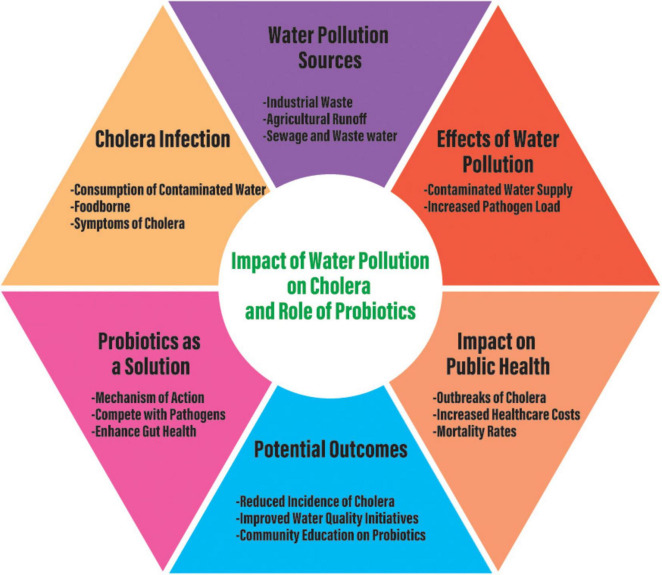
Conceptual diagram regarding cholera infection due to water pollution and the role of probiotics to solve cholera infection to save human life.

The potential for probiotics to provide long-term protection against cholera has also been demonstrated. For example, *Bacillus velezensis* used in aquaculture showed not only antimicrobial activity against *V. cholerae*, but also enhancement of host immune response. This dual role in enhancing the frontline antimicrobial defense and boosting long-term immunity suggests that probiotics may useful in broader cholera control programs, particularly in regions where cholera is endemic and where maintaining long-term immunity is crucial (Zhu et al., 2021). Despite these promising findings, translating laboratory and animal model successes to human clinical trials has been inconsistent. Probiotic strains derived from healthy children showed inhibitory effects on Vibrio biofilm formation and adhesion, yet the antibacterial activity against *V. parahaemolyticus* biofilms was limited. These observations highlight the need for further research to identify robust, strain-specific probiotics for cholera prevention and treatment (Kaur et al., 2018). Probiotics can also play a role in environmental management for preventing cholera outbreaks. Certain probiotics can disrupt *V. cholerae* biofilms in natural water bodies, and thereby reduce the environmental reservoirs of the pathogen (Kaur et al., 2018). By targeting *V. cholerae* in its natural habitats, probiotics can help limit the spread of *V. cholerae* from environmental sources, and thus provide an additional layer of protection in communities where waterborne transmission is a major concern. After studying several articles, we developed a concept that can create a solution to decrease cholera infection in those countries under cholera infection and probiotics should be a solution to decrease this infection (Figure 2).

## 6 Conclusion

Driven largely by water pollution and conditions of inadequate sanitation, cholera continues to be a significant public health challenge in Bangladesh. The intricate link between environmental factors, such as water pollution, and cholera outbreaks underscores the need for comprehensive strategies to manage and prevent the disease. The traditional methods of managing cholera, including ORT, antibiotics, and vaccination programs, have proven effective, but they are not without limitations. The emergence of antibiotic resistance, logistical challenges in vaccine distribution, and the recurring nature of outbreaks highlight the need for novel and complementary approaches. Probiotics offer a promising avenue for cholera prevention and management. They provide beneficial effects against cholera through various mechanisms, such as the competitive exclusion of the pathogen, enhancement of the gut barrier function, modulation of immune responses, and disruption of pathogen signaling. The development of engineered probiotics may further expand their usefulness, and provide innovative solutions that may enable us to detect and respond to specific pathogenic signals in real time. Additionally, the integration of probiotics with existing interventions, such as vaccines and ORS, can enhance the overall efficacy of cholera control measures. In addressing the cholera epidemic, it is crucial to consider the population groups most affected by this disease. Vulnerable groups, particularly young children and elderly individuals, are often at higher risk due to their weaker immune systems. Additionally, occupations that involve exposure to contaminated water sources, such as fishermen and agricultural workers, should be prioritized in public health interventions. A better understanding of these demographics can guide targeted strategies for prevention and management. Recent studies emphasize the potential of specific probiotic strains, such as *Lactobacillus rhamnosus* GG and *Saccharomyces boulardii*, in combating diarrheal diseases, including cholera. Combining probiotics with established interventions like ORS and vaccines could enhance their efficacy by improving gut health and boosting immune responses. Additionally, research on mechanisms like quorum sensing disruption and biofilm inhibition can drive the development of engineered probiotics with targeted action against *V. cholerae*. Long-term clinical trials and innovative strategies, such as using probiotics to neutralize contaminated water sources, are essential to validate their application in endemic regions like Bangladesh. To ensure practical application, it is essential to propose targeted policy recommendations tailored to the specific conditions of Bangladesh.

Cholera can be managed through immediate measures like emergency response centers, safe water supply, hygiene promotion, vaccination, isolation, and long-term strategies. Long-term measures include improving sanitation, investing in modern water treatment plants, promoting community-led sanitation, and enhancing water quality. Communicating cholera effectively requires a coordinated approach integrating immediate containment with long-term preventive measures. Research should focus on identifying gaps in sanitation and water quality management while fostering innovative solutions tailored to the needs of vulnerable populations. Collaboration among governments, NGOs, and local communities is crucial for sustainable health outcomes. Policies should focus on improving access to clean water and sanitation facilities, increasing awareness of probiotics’ role in public health, and encouraging partnerships between researchers, policymakers, and community stakeholders. Furthermore, investment in infrastructure for the production and distribution of probiotics could facilitate their accessibility and affordability. By harnessing the natural protective effects of probiotics and integrating them into broader cholera control strategies, more sustainable and effective measures can be established. These measures would not only reduce the disease burden but also contribute to improving overall public health outcomes in vulnerable populations. The ongoing exploration of probiotics as a complementary tool in cholera prevention holds immense potential for addressing this persistent public health challenge in Bangladesh.

## References

[B1] Ahmed K. Rahman A. Sarkar M. Islam J. Jahan I. Moniruzzaman M. (2016). Assessment on the level of contamination of Turag river at Tongi area in Dhaka, Bangladesh. *J. Sci. Ind. Res.* 51 193–202. 10.3329/bjsir.v51i3.29431

[B2] Akanda A. S. Jutla A. S. Gute D. M. Sack R. B. Alam M. Huq A. (2013). Population vulnerability to biannual cholera outbreaks and associated macro-scale drivers in the Bengal Delta. *Am. J. Trop. Med. Hyg.* 89 950–959. 10.4269/ajtmh.12-0492 24019441 PMC3820342

[B3] Alam M. Islam M. T. Rashed S. M. Johura F. T. Bhuiyan N. A. Delgado G. (2012). *Vibrio cholerae* classical biotype strains reveal distinct signatures in Mexico. *J. Clin. Microbiol.* 50 2212–2216. 10.1128/jcm.00189-12 22518867 PMC3405568

[B4] Alamdary S. Z. Bakhshi B. (2020). Lactobacillus acidophilus attenuates toxin production by *Vibrio cholerae* and *shigella* dysenteriae following intestinal epithelial cells infection. *Microb. Pathog.* 149:104543. 10.1016/j.micpath.2020.104543 33010360

[B5] Albert M. J. Qadri F. Wahed M. A. Ahmed T. Rahman A. S. Ahmed F. (2003). Supplementation with zinc, but not vitamin A, improves seroconversion to vibriocidal antibody in children given an oral cholera vaccine. *J. Infect. Dis.* 187 909–913. 10.1086/368132 12660937

[B6] Almagro-Moreno S. Pruss K. Taylor R. K. (2015). Intestinal colonization dynamics of *Vibrio cholerae*. *PLoS Pathog.* 11:e1004787. 10.1371/journal.ppat.1004787 25996593 PMC4440752

[B7] Amdekar S. Dwivedi D. Roy P. Kushwah S. Singh V. (2010). Probiotics: multifarious oral vaccine against infectious traumas. *FEMS Immunol. Med. Microbiol.* 58 299–306. 10.1111/j.1574-695X.2009.00630.x 20100178

[B8] Amrofell M. B. Rottinghaus A. G. Moon T. S. (2020). Engineering microbial diagnostics and therapeutics with smart control. *Curr. Opin. Biotechnol.* 66 11–17. 10.1016/j.copbio.2020.05.006 32563763 PMC7744387

[B9] Anawar H. M. Safiullah S. Yoshioka T. (2000). Environmental exposure assessment of Chromium and other tannery pollutants at Hazaribagh area, Dhaka, Bangladesh, and health risk. *J. Environ. Chem.* 10 549–556. 10.5985/jec.10.549

[B10] Anik A. H. Khan R. Hossain S. Siddique M. A. B. Tamim U. Islam A. (2022). Reconciling the geogenic and non-crustal origins of elements in an Indo-Bangla transboundary river, Atrai: pollution status, sediment quality, and preliminary risk assessment. *Environ. Res.* 214:114134. 10.1016/j.envres.2022.114134 35998696

[B11] Azad M. A. K. Sarker M. Wan D. (2018). Immunomodulatory effects of probiotics on cytokine profiles. *Biomed. Res. Int.* 2018:8063647. 10.1155/2018/8063647 30426014 PMC6218795

[B12] Azman A. S. Lessler J. Satter S. M. McKay M. V. Khan A. Ahmed D. (2015). Tracking cholera through surveillance of oral rehydration solution sales at pharmacies: insights from Urban Bangladesh. *PLoS Negl. Trop. Dis.* 9:e0004230. 10.1371/journal.pntd.0004230 26641649 PMC4671575

[B13] Azman A. S. Rudolph K. E. Cummings D. A. Lessler J. (2013). The incubation period of cholera: a systematic review. *J. Infect.* 66 432–438. 10.1016/j.jinf.2012.11.013 23201968 PMC3677557

[B14] Basumatary C. Kaur R. Kaur S. (2021). Treatment strategies of cholera: a review. *Eur. J. Transl. Clin. Med.* 7:2020.

[B15] Bermúdez-Humarán L. G. Salinas E. Ortiz G. G. Ramirez-Jirano L. J. Morales J. A. Bitzer-Quintero O. K. (2019). From probiotics to psychobiotics: live beneficial bacteria which act on the brain-gut axis. *Nutrients* 11:890. 10.3390/nu11040890 31010014 PMC6521058

[B16] Bhandare S. Colom J. Baig A. Ritchie J. M. Bukhari H. Shah M. A. (2019). Reviving phage therapy for the treatment of cholera. *J. Infect. Dis.* 219 786–794. 10.1093/infdis/jiy563 30395214 PMC7610978

[B17] Bilal H. Li X. Iqbal M. S. Mu Y. Tulcan R. X. S. Ghufran M. A. (2023). Surface water quality, public health, and ecological risks in Bangladesh-a systematic review and meta-analysis over the last two decades. *Environ. Sci. Pollut. Res. Int.* 30 91710–91728. 10.1007/s11356-023-28879-x 37526829

[B18] Boyd E. F. Waldor M. K. (2002). Evolutionary and functional analyses of variants of the toxin-coregulated pilus protein TcpA from toxigenic *Vibrio cholerae* non-O1/non-O139 serogroup isolates. *Microbiology* 148 1655–1666. 10.1099/00221287-148-6-1655 12055286

[B19] Castro-Herrera V. M. Rasmussen C. Wellejus A. Miles E. A. Calder P. C. (2020). *In vitro* effects of live and heat-inactivated bifidobacterium animalis Subsp. Lactis, BB-12 and Lactobacillus rhamnosus GG on Caco-2 Cells. *Nutrients* 12:1719. 10.3390/nu12061719 32521765 PMC7352502

[B20] Chakraborti D. Rahman M. M. Das B. Murrill M. Dey S. Chandra Mukherjee S. (2010). Status of groundwater arsenic contamination in Bangladesh: a 14-year study report. *Water Res.* 44 5789–5802. 10.1016/j.watres.2010.06.051 20684969

[B21] Chen L. Gu Q. Li P. Li Y. Song D. Yang J. (2018). Purification and characterization of plantaricin ZJ316, a novel bacteriocin against listeria monocytogenes from Lactobacillus plantarum ZJ316. *J. Food Prot.* 81 1929–1935. 10.4315/0362-028x.Jfp-18-306 30427729

[B22] Chettri U. Joshi S. R. (2022). A first calibration of culturable bacterial diversity and their dual resistance to heavy metals and antibiotics along altitudinal zonation of the Teesta River. *Arch. Microbiol.* 204:241. 10.1007/s00203-022-02858-1 35378604

[B23] Chhabra R. (2021). “Irrigation water: quality criteria,” in *Salt-Affected Soils and Marginal Waters: Global Perspectives and Sustainable Management*, (Cham: Springer), 431–486. Available online at: https://link.springer.com/book/10.1007/978-3-030-78435-5

[B24] Chopra A. K. Sharma M. K. Chamoli S. (2011). Bioaccumulation of organochlorine pesticides in aquatic system–an overview. *Environ. Monit. Assess.* 173 905–916. 10.1007/s10661-010-1433-4 20306340

[B25] Chowdhury F. Aziz A. B. Ahmmed F. Ahmed T. Kang S. S. Im J. (2023). The interplay between WASH practices and vaccination with oral cholera vaccines in protecting against cholera in urban Bangladesh: reanalysis of a cluster-randomized trial. *Vaccine* 41 2368–2375. 10.1016/j.vaccine.2023.02.054 36898931 PMC10102718

[B26] Chowdhury F. Ross A. G. Islam M. T. McMillan N. A. J. Qadri F. (2022). Diagnosis, management, and future control of cholera. *Clin. Microbiol. Rev.* 35:e0021121. 10.1128/cmr.00211-21 35726607 PMC9491185

[B27] Chowdhury M. D. A. Billah T. Rahman M. R. Bakri M. K. B. Barua S. Morshed A. (2024). Evaluation of water quality indexes and heavy metal pollution indexes of different industrial effluents and Karnaphuli river water in Chattogram, Bangladesh. *Environ. Qual. Manag.* 34:e22290. 10.1002/tqem.22290

[B28] Cristofori F. Dargenio V. N. Dargenio C. Miniello V. L. Barone M. Francavilla R. (2021). Anti-inflammatory and immunomodulatory effects of probiotics in gut inflammation: a door to the body. *Front. Immunol.* 12:578386. 10.3389/fimmu.2021.578386 33717063 PMC7953067

[B29] Cruz K. C. P. Enekegho L. O. Stuart D. T. (2022). Bioengineered probiotics: synthetic biology can provide live cell therapeutics for the treatment of foodborne diseases. *Front. Bioeng. Biotechnol.* 10:890479. 10.3389/fbioe.2022.890479 35656199 PMC9152101

[B30] Czerucka D. Piche T. Rampal P. (2007). Review article: yeast as probiotics – Saccharomyces boulardii. *Aliment. Pharmacol. Ther.* 26 767–778. 10.1111/j.1365-2036.2007.03442.x 17767461

[B31] Davies H. G. Bowman C. Luby S. P. (2017). Cholera - management and prevention. *J. Infect.* 74 (Suppl. 1), S66–S73. 10.1016/s0163-4453(17)30194-9 28646965

[B32] De Haan L. Hirst T. R. (2004). Cholera toxin: a paradigm for multi-functional engagement of cellular mechanisms (Review). *Mol. Membr. Biol.* 21 77–92. 10.1080/09687680410001663267 15204437

[B33] de Magny G. C. Mozumder P. K. Grim C. J. Hasan N. A. Naser M. N. Alam M. (2011). Role of zooplankton diversity in *Vibrio cholerae* population dynamics and in the incidence of cholera in the Bangladesh Sundarbans. *Appl. Environ. Microbiol.* 77 6125–6132. 10.1128/aem.01472-10 21764957 PMC3165371

[B34] Dimitrov D. T. Troeger C. Halloran M. E. Longini I. M. Chao D. L. (2014). Comparative effectiveness of different strategies of oral cholera vaccination in Bangladesh: a modeling study. *PLoS Negl. Trop. Dis.* 8:e3343. 10.1371/journal.pntd.0003343 25473851 PMC4256212

[B35] du Plessis A. (ed.) (2023). “Water resources from a global perspective,” in *South Africa’s Water Predicament: Freshwater’s Unceasing Decline*, (Cham: Springer), 1–25.

[B36] Everard A. Cani P. D. (2014). Gut microbiota and GLP-1. *Rev. Endocr. Metab. Disord.* 15 189–196. 10.1007/s11154-014-9288-6 24789701

[B37] Flura M. A. Akhery N. Mohosena B. Masud H. (2016). Physico-chemical and biological properties of water from the river Meghna, Bangladesh. *Int. J. Fish Aquat. Stud.* 4 161–165.

[B38] Focareta A. Paton J. C. Morona R. Cook J. Paton A. W. (2006). A recombinant probiotic for treatment and prevention of cholera. *Gastroenterology* 130 1688–1695. 10.1053/j.gastro.2006.02.005 16697733

[B39] Gani M. A. Sajib A. M. Siddik M. A. Md M. (2023). Assessing the impact of land use and land cover on river water quality using water quality index and remote sensing techniques. *Environ. Monit. Assess.* 195:449. 10.1007/s10661-023-10989-1 36882593

[B40] Gou H. Z. Zhang Y. L. Ren L. F. Li Z. J. Zhang L. (2022). How do intestinal probiotics restore the intestinal barrier? *Front. Microbiol.* 13:929346. 10.3389/fmicb.2022.929346 35910620 PMC9330398

[B41] Grant S. L. Tamason C. C. Hoque B. A. Jensen P. K. (2015). Drinking cholera: salinity levels and palatability of drinking water in coastal Bangladesh. *Trop. Med. Int. Health* 20 455–461. 10.1111/tmi.12455 25581714

[B42] Gupta V. Garg R. (2009). Probiotics. *Indian J. Med. Microbiol.* 27 202–209. 10.4103/0255-0857.53201 19584499

[B43] Hasan M. K. Shahriar A. Jim K. U. (2019). Water pollution in Bangladesh and its impact on public health. *Heliyon* 5:e02145. 10.1016/j.heliyon.2019.e02145 31406938 PMC6684462

[B44] Hassan K. T. Ferdoushi Z. Rana M. M. Alam M. S. (2024). Assessing the seasonal variability of water quality and heavy metals concentration in sediment, water, and fish muscles of Korotoa River in Bangladesh. *Aquac. Res.* 2024:5343363. 10.1155/2024/5343363

[B45] Hassan M. M. Ahmed S. A. Rahman K. A. Biswas T. K. (2008). Pattern of medical waste management: existing scenario in Dhaka City, Bangladesh. *BMC Public Health* 8:36. 10.1186/1471-2458-8-36 18221548 PMC2254398

[B46] Hegde S. T. Lee E. C. Islam Khan A. Lauer S. A. Islam M. T. Rahman Bhuiyan T. (2021). Clinical cholera surveillance sensitivity in Bangladesh and implications for large-scale disease control. *J. Infect. Dis.* 224 S725–S731. 10.1093/infdis/jiab418 34453539 PMC8687068

[B47] Holmgren J. (2021a). Modern history of cholera vaccines and the pivotal role of icddr,b. *J. Infect. Dis.* 224 S742–S748. 10.1093/infdis/jiab423 34453544 PMC8687080

[B48] Holmgren J. (2021b). An update on cholera immunity and current and future cholera vaccines. *Trop. Med. Infect. Dis.* 6:64. 10.3390/tropicalmed6020064 33925118 PMC8167659

[B49] Hossain L. Sarker S. K. Khan M. S. (2018). Evaluation of present and future wastewater impacts of textile dyeing industries in Bangladesh. *Environ. Dev.* 26 23–33. 10.1016/j.envdev.2018.03.005

[B50] Hsiao A. Ahmed A. M. Subramanian S. Griffin N. W. Drewry L. L. Petri W. A. Jr. (2014). Members of the human gut microbiota involved in recovery from *Vibrio cholerae* infection. *Nature* 515 423–426. 10.1038/nature13738 25231861 PMC4353411

[B51] Hsueh B. Y. Waters C. M. (2019). Combating cholera. *F1000Res* 8 F1000 Faculty Rev-589. 10.12688/f1000research.18093.1 31069064 PMC6492228

[B52] Huq A. Sack R. B. Nizam A. Longini I. M. Nair G. B. Ali A. (2005). Critical factors influencing the occurrence of *Vibrio cholerae* in the environment of Bangladesh. *Appl. Environ. Microbiol.* 71 4645–4654. 10.1128/aem.71.8.4645-4654.2005 16085859 PMC1183289

[B53] Huq A. West P. A. Small E. B. Huq M. I. Colwell R. R. (1984). Influence of water temperature, salinity, and pH on survival and growth of toxigenic *Vibrio cholerae* serovar 01 associated with live copepods in laboratory microcosms. *Appl. Environ. Microbiol.* 48 420–424. 10.1128/aem.48.2.420-424.1984 6486784 PMC241529

[B54] Huq M. E. Fahad S. Shao Z. Sarven M. S. Khan I. A. Alam M. (2020). Arsenic in a groundwater environment in Bangladesh: occurrence and mobilization. *J. Environ. Manage.* 262:110318. 10.1016/j.jenvman.2020.110318 32250801

[B55] Islam M. N. Ahmed M. J. Hossain M. A. Siraj S. (2013). Physicochemical assessment of water pollutants due to the ship breaking activities and its impact on the coastal environment of Chittagong, Bangladesh. *Eur. Chem. Bull.* 2 1053–1059. 10.17628/ECB.2013.2.975

[B56] Islam M. R. Das N. Barua P. Hossain M. B. Venkatramanan S. Chung S. (2017). Environmental assessment of water and soil contamination in Rajakhali Canal of Karnaphuli River (Bangladesh) impacted by anthropogenic influences: a preliminary case study. *Appl. Water Sci.* 7 997–1010. 10.1007/s13201-015-0310-2

[B57] Islam S. M. D. Mondal P. K. Ojong N. Bodrud-Doza M. Siddique M. A. B. Hossain M. (2021). Water, sanitation, hygiene and waste disposal practices as COVID-19 response strategy: insights from Bangladesh. *Environ. Dev. Sustain.* 23 11953–11974. 10.1007/s10668-020-01151-9 33424423 PMC7778416

[B58] Islam S. Azam G. (2015). Seasonal variation of physicochemical and toxic properties in three major rivers; Shitalakhya, Buriganga and Turag around Dhaka city, Bangladesh. *J. Bio. Environ. Sci.* 7 120–131.

[B59] Ivers L. C. (2018). Advancing control of cholera in the interest of the most vulnerable in our global society. *J. Infect. Dis.* 218 S135–S136. 10.1093/infdis/jiy458 30184099 PMC6188543

[B60] Jimenez A. G. Sperandio V. (2019). “Quorum sensing and the gut microbiome,” in *Quorum Sensing*, ed. Tommonaro G. (Amsterdam: Elsevier), 151–169.

[B61] Jutla A. Whitcombe E. Hasan N. Haley B. Akanda A. Huq A. (2013). Environmental factors influencing epidemic cholera. *Am. J. Trop. Med. Hyg.* 89 597–607. 10.4269/ajtmh.12-0721 23897993 PMC3771306

[B62] Karn S. K. Harada H. (2001). Surface water pollution in three urban territories of Nepal, India, and Bangladesh. *Environ. Manage.* 28 483–496. 10.1007/s002670010238 11494067

[B63] Kaur S. Kaur R. Rani N. Sharma S. Joshi M. (2021). “Sources and selection criteria of probiotics,” in *Advances in Probiotics for Sustainable Food and Medicine. Microorganisms for Sustainability*, eds Goel G. Kumar A. (Singapore: Springer), 27–43. 10.1007/978-981-15-6795-7_2

[B64] Kaur S. Sharma P. Kalia N. Singh J. Kaur S. (2018). Anti-biofilm properties of the fecal probiotic lactobacilli against Vibrio spp. *Front. Cell Infect. Microbiol.* 8:120. 10.3389/fcimb.2018.00120 29740541 PMC5928150

[B65] Khan I. A. Khan A. I. Rahman A. Siddique S. A. Islam M. T. Bhuiyan M. A. I. (2019). Organization and implementation of an oral cholera vaccination campaign in an endemic urban setting in Dhaka, Bangladesh. *Glob. Health Action* 12:1574544. 10.1080/16549716.2019.1574544 30764750 PMC6383613

[B66] Kibria G. Yousuf Haroon A. (2017). “Climate change impacts on wetlands of Bangladesh, its biodiversity and ecology, and actions and programs to reduce risks,” in *Wetland Science Perspectives South Asia*, eds Prusty B. Chandra R. Azeez P. (New Delhi: Springer), 189–204. 10.1007/978-81-322-3715-0_10

[B67] Knappett P. S. Escamilla V. Layton A. McKay L. D. Emch M. Williams D. E. (2011). Impact of population and latrines on fecal contamination of ponds in rural Bangladesh. *Sci. Total Environ.* 409 3174–3182. 10.1016/j.scitotenv.2011.04.043 21632095 PMC3150537

[B68] Kunze W. A. Mao Y. K. Wang B. Huizinga J. D. Ma X. Forsythe P. (2009). Lactobacillus reuteri enhances excitability of colonic AH neurons by inhibiting calcium-dependent potassium channel opening. *J. Cell Mol. Med.* 13 2261–2270. 10.1111/j.1582-4934.2009.00686.x 19210574 PMC6529958

[B69] Lantagne D. Yates T. (2018). Household water treatment and cholera control. *J. Infect. Dis.* 218 S147–S153. 10.1093/infdis/jiy488 30215739 PMC6188534

[B70] Lei H. Sheng Z. Ding Q. Chen J. Wei X. Lam D. M. (2011). Evaluation of oral immunization with recombinant avian influenza virus HA1 displayed on the Lactococcus lactis surface and combined with the mucosal adjuvant cholera toxin subunit B. *Clin. Vaccine Immunol.* 18 1046–1051. 10.1128/cvi.00050-11 21632890 PMC3147322

[B71] Leibovici-Weissman Y. A. Neuberger A. Bitterman R. Sinclair D. Salam M. A. Paul M. (2014). Antimicrobial drugs for treating cholera. *Cochr. Datab. Syst. Rev*. 2014:CD008625. 10.1002/14651858.CD008625.pub2 24944120 PMC4468928

[B72] Lenoir-Wijnkoop I. Sanders M. E. Cabana M. D. Caglar E. Corthier G. Rayes N. (2007). Probiotic and prebiotic influence beyond the intestinal tract. *Nutr. Rev.* 65 469–489. 10.1111/j.1753-4887.2007.tb00272.x 18038940

[B73] Liberato S. C. Singh G. Mulholland K. (2015). Zinc supplementation in young children: a review of the literature focusing on diarrhoea prevention and treatment. *Clin. Nutr.* 34 181–188. 10.1016/j.clnu.2014.08.002 25176404

[B74] Lutz C. Erken M. Noorian P. Sun S. McDougald D. (2013). Environmental reservoirs and mechanisms of persistence of *Vibrio cholerae*. *Front. Microbiol.* 4:375. 10.3389/fmicb.2013.00375 24379807 PMC3863721

[B75] Majed N. Islam M. A. S. (2022). Contaminant discharge from outfalls and subsequent aquatic ecological risks in the river systems in Dhaka city: extent of waste load contribution in pollution. *Front. Public Health* 10:880399. 10.3389/fpubh.2022.880399 35692332 PMC9177986

[B76] Mao N. Cubillos-Ruiz A. Cameron D. E. Collins J. J. (2018). Probiotic strains detect and suppress cholera in mice. *Sci. Transl. Med.* 10:eaao2586. 10.1126/scitranslmed.aao2586 29899022 PMC7821980

[B77] Matsuda F. Chowdhury M. I. Saha A. Asahara T. Nomoto K. Tarique A. A. (2011). Evaluation of a probiotics, Bifidobacterium breve BBG-01, for enhancement of immunogenicity of an oral inactivated cholera vaccine and safety: a randomized, double-blind, placebo-controlled trial in Bangladeshi children under 5 years of age. *Vaccine* 29 1855–1858. 10.1016/j.vaccine.2010.12.133 21236234

[B78] McCarthy S. A. (1996). Effects of temperature and salinity on survival of toxigenic*Vibrio cholerae* O1 in seawater. *Microb. Ecol.* 31 167–175. 10.1007/bf00167862 24185740

[B79] Mina S. A. Marzan L. W. Sultana T. Akter Y. (2018). Quality assessment of commercially supplied drinking jar water in Chittagong City, Bangladesh. *Appl. Water Sci.* 8 1–8.

[B80] Monir M. M. Hossain T. Morita M. Ohnishi M. Johura F. T. Sultana M. (2022). Genomic characteristics of recently recognized *Vibrio cholerae* El Tor lineages associated with cholera in Bangladesh, 1991 to 2017. *Microbiol. Spectr.* 10:e0039122. 10.1128/spectrum.00391-22 35315699 PMC9045249

[B81] Mourshed M. Masud M. H. Rashid F. Joardder M. U. H. (2017). Towards the effective plastic waste management in Bangladesh: a review. *Environ. Sci. Pollut. Res. Int.* 24 27021–27046. 10.1007/s11356-017-0429-9 29079979

[B82] Mukherjee D. Chattopadhyay M. Lahiri S. (1993). Water quality of the River Ganga (The Ganges) and some of its physico-chemical properties. *Environmentalist* 13 199–210. 10.1007/BF01901382

[B83] Nag D. Breen P. Raychaudhuri S. Withey J. H. (2018). Glucose metabolism by *Escherichia coli* inhibits *Vibrio cholerae* intestinal colonization of zebrafish. *Infect. Immun.* 86 e00486–18. 10.1128/iai.00486-18 30249745 PMC6246912

[B84] Nagpal R. Kumar M. Yadav A. K. Hemalatha R. Yadav H. Marotta F. (2016). Gut microbiota in health and disease: an overview focused on metabolic inflammation. *Benef. Microbes* 7 181–194. 10.3920/bm2015.0062 26645350

[B85] Nalin D. (2021). Issues and controversies in the evolution of Oral Rehydration Therapy (ORT). *Trop. Med. Infect. Dis.* 6:34. 10.3390/tropicalmed6010034 33809275 PMC8005945

[B86] Nandakumar N. S. Pugazhendhi S. Madhu Mohan K. Jayakanthan K. Ramakrishna B. S. (2009). Effect of *Vibrio cholerae* on chemokine gene expression in HT29 cells and its modulation by Lactobacillus GG. *Scand. J. Immunol.* 69 181–187. 10.1111/j.1365-3083.2008.02214.x 19281529

[B87] Nguyen T. T. Kim C. Goucher G. Kim J.-H. (2023). Associations of water quality with cholera in case-control studies: a systematic review and meta-analysis. *medRxiv [Preprint]* 10.1101/2023.09.06.23295113 PMC10464135 37644449

[B88] Ondrasek G. (2013). “Water scarcity and water stress in agriculture,” in *Physiological Mechanisms and Adaptation Strategies in Plants Under Changing Environment*, Vol. 1 eds Ahmad P. Wani M. R. (Berlin: Springer), 75–96.

[B89] Osiemo M. M. Ogendi G. M. M’Erimba C. (2019). Microbial quality of drinking water and prevalence of water-related diseases in Marigat Urban Centre, Kenya. *Environ. Health Insights* 13:1178630219836988. 10.1177/1178630219836988 30899150 PMC6419249

[B90] Pandey P. K. Kass P. H. Soupir M. L. Biswas S. Singh V. P. (2014). Contamination of water resources by pathogenic bacteria. *AMB Express* 4:51. 10.1186/s13568-014-0051-x 25006540 PMC4077002

[B91] Plaza-Diaz J. Ruiz-Ojeda F. J. Gil-Campos M. Gil A. (2019). Mechanisms of action of probiotics. *Adv. Nutr.* 10 S49–S66. 10.1093/advances/nmy063 30721959 PMC6363529

[B92] Rahman M. A. Islam M. R. Kumar S. Al-Reza S. M. (2021). Drinking water quality, exposure and health risk assessment for the school-going children at school time in the southwest coastal of Bangladesh. *J. Water Sanit. Hyg. Dev.* 11 612–628. 10.2166/washdev.2021.016

[B93] Reid G. (1999). The scientific basis for probiotic strains of Lactobacillus. *Appl. Environ. Microbiol.* 65 3763–3766. 10.1128/aem.65.9.3763-3766.1999 10473372 PMC99697

[B94] Reid G. Anand S. Bingham M. O. Mbugua G. Wadstrom T. Fuller R. (2005). Probiotics for the developing world. *J. Clin. Gastroenterol.* 39 485–488. 10.1097/01.mcg.0000165648.32371.38 15942433

[B95] Resta-Lenert S. Barrett K. E. (2003). Live probiotics protect intestinal epithelial cells from the effects of infection with enteroinvasive *Escherichia coli* (EIEC). *Gut* 52 988–997. 10.1136/gut.52.7.988 12801956 PMC1773702

[B96] Reza A. Yousuf T. B. (2016). Impacts of waste dumping on water quality in the Buriganga River, Bangladesh and possible mitigation measures. *J. Environ.* 11 35–40.

[B97] Ronsmans C. Bennish M. L. Wierzba T. (1988). Diagnosis and management of dysentery by community health workers. *Lancet* 2 552–555. 10.1016/s0140-6736(88)92669-4 2900931

[B98] Saima S. Ferdous J. Sultana R. Rashid R. B. Almeida S. Begum A. (2023). Detecting enteric pathogens in low-risk drinking water in Dhaka, Bangladesh: an assessment of the WHO water safety categories. *Trop. Med. Infect. Dis.* 8:321. 10.3390/tropicalmed8060321 37368739 PMC10302631

[B99] Sarkar A. M. Rahman A. Samad A. Bhowmick A. C. Islam J. B. (2019). Surface and ground water pollution in Bangladesh: a review. *Asian Rev. Environ. Earth Sci.* 6 47–69.

[B100] Sigman M. Luchette F. A. (2012). Cholera: something old, something new. *Surg. Infect.* 13 216–222. 10.1089/sur.2012.127 22913779

[B101] Singh K. P. Mohan D. Sinha S. Dalwani R. (2004). Impact assessment of treated/untreated wastewater toxicants discharged by sewage treatment plants on health, agricultural, and environmental quality in the wastewater disposal area. *Chemosphere* 55 227–255. 10.1016/j.chemosphere.2003.10.050 14761695

[B102] Sit B. Zhang T. Fakoya B. Akter A. Biswas R. Ryan E. T. (2019). Oral immunization with a probiotic cholera vaccine induces broad protective immunity against *Vibrio cholerae* colonization and disease in mice. *PLoS Negl. Trop. Dis.* 13:e0007417. 10.1371/journal.pntd.0007417 31150386 PMC6561597

[B103] Śliżewska K. Markowiak-Kopeæ P. Śliżewska W. (2020). The role of probiotics in cancer prevention. *Cancers* 13:120. 10.3390/cancers13010020 33374549 PMC7793079

[B104] Tejada-Simon M. V. Lee J. H. Ustunol Z. Pestka J. J. (1999). Ingestion of yogurt containing Lactobacillus acidophilus and Bifidobacterium to potentiate immunoglobulin A responses to cholera toxin in mice. *J. Dairy Sci.* 82 649–660. 10.3168/jds.S0022-0302(99)75281-1 10212452

[B105] Tejero-Sariñena S. Barlow J. Costabile A. Gibson G. R. Rowland I. (2012). *In vitro* evaluation of the antimicrobial activity of a range of probiotics against pathogens: evidence for the effects of organic acids. *Anaerobe* 18 530–538. 10.1016/j.anaerobe.2012.08.004 22959627

[B106] Tette F. M. Kwofie S. K. Wilson M. D. (2022). Therapeutic anti-depressant potential of microbial GABA Produced by Lactobacillus rhamnosus strains for GABAergic signaling restoration and inhibition of addiction-induced HPA axis hyperactivity. *Curr. Issues Mol. Biol.* 44 1434–1451. 10.3390/cimb44040096 35723354 PMC9164062

[B107] Tong J. Satyanarayanan S. K. Su H. (2020). Nutraceuticals and probiotics in the management of psychiatric and neurological disorders: a focus on microbiota-gut-brain-immune axis. *Brain Behav. Immun.* 90 403–419. 10.1016/j.bbi.2020.08.027 32889082

[B108] Turroni F. Milani C. Ventura M. van Sinderen D. (2022). The human gut microbiota during the initial stages of life: insights from bifidobacteria. *Curr. Opin. Biotechnol.* 73 81–87. 10.1016/j.copbio.2021.07.012 34333445

[B109] Uddin M. J. Jeong Y. K. (2021). Urban river pollution in Bangladesh during last 40 years: potential public health and ecological risk, present policy, and future prospects toward smart water management. *Heliyon* 7:e06107. 10.1016/j.heliyon.2021.e06107 33659727 PMC7892934

[B110] Uddin M. J. Wahed T. Saha N. C. Kaukab S. S. Khan I. A. Khan A. I. (2014). Coverage and acceptability of cholera vaccine among high-risk population of urban Dhaka, Bangladesh. *Vaccine* 32 5690–5695. 10.1016/j.vaccine.2014.08.021 25149429

[B111] Usmani M. Brumfield K. D. Jamal Y. Huq A. Colwell R. R. Jutla A. (2021). A review of the environmental trigger and transmission components for prediction of cholera. *Trop. Med. Infect. Dis.* 6:147. 10.3390/tropicalmed6030147 PMC8396309 34449728

[B112] Versalovic J. Iyer C. Ping Lin Y. Huang Y. Dobrogosz W. (2008). Commensal-derived probiotics as anti-inflammatory agents. *Microb. Ecol. Health Dis.* 20 86–93. 10.1080/08910600802106491

[B113] VidyaLaxme B. Rovetto A. Grau R. Agrawal R. (2014). Synergistic effects of probiotic Leuconostoc mesenteroides and Bacillus subtilis in malted ragi (Eleucine corocana) food for antagonistic activity against V. cholerae and other beneficial properties. *J. Food Sci. Technol.* 51 3072–3082. 10.1007/s13197-012-0834-5 26396299 PMC4571230

[B114] Weil A. A. Begum Y. Chowdhury F. Khan A. I. Leung D. T. LaRocque R. C. (2014). Bacterial shedding in household contacts of cholera patients in Dhaka, Bangladesh. *Am. J. Trop. Med. Hyg.* 91 738–742. 10.4269/ajtmh.14-0095 25114012 PMC4183396

[B115] Wieërs G. Belkhir L. Enaud R. Leclercq S. Philippart de Foy J. M. Dequenne I. (2019). How probiotics affect the microbiota. *Front. Cell Infect. Microbiol.* 9:454. 10.3389/fcimb.2019.00454 32010640 PMC6974441

[B116] Yu R. K. Usuki S. Itokazu Y. Wu H. C. (2016). Novel GM1 ganglioside-like peptide mimics prevent the association of cholera toxin to human intestinal epithelial cells *in vitro*. *Glycobiology* 26 63–73. 10.1093/glycob/cwv080 26405107 PMC4672148

[B117] Zamri H. F. Shamsudin M. N. Rahim R. A. Neela V. (2012). Oral vaccination with Lactococcus lactis expressing the *Vibrio cholerae* Wzm protein to enhance mucosal and systemic immunity. *Vaccine* 30 3231–3238. 10.1016/j.vaccine.2012.02.012 22426330

[B118] Zhu X. Zhang S. Zhou L. Ao S. Tang H. Zhou Y. (2021). Probiotic potential of Bacillus velezensis: antimicrobial activity against non-O1 *Vibrio cholerae* and immune enhancement effects on Macrobrachium nipponense. *Aquaculture* 541:736817. 10.1016/j.aquaculture.2021.736817

[B119] Zohura F. Thomas E. D. Masud J. Bhuyian M. S. I. Parvin T. Monira S. (2022). Formative research for the development of the CHoBI7 cholera rapid response program for cholera hotspots in Bangladesh. *Int. J. Environ. Res. Public Health* 19:13352. 10.3390/ijerph192013352 36293930 PMC9603179

